# Integrating Neglected Tropical Disease and Immunization Programs: The Experiences of the Tanzanian Ministry of Health

**DOI:** 10.4269/ajtmh.15-0724

**Published:** 2016-09-07

**Authors:** Upendo John Mwingira, Arianna Rubin Means, Maria Chikawe, Bernard Kilembe, Dafrossa Lyimo, Kathryn Crowley, Neema Rusibamayila, Andreas Nshala, Alex Mphuru

**Affiliations:** ^1^ Neglected Tropical Disease Control Programme, Ministry of Health, Community Development, Gender, Elderly and Children, Dar es Salaam, Tanzania; ^2^ Department of Global Health, University of Washington, Seattle, Washington; ^3^ Immunization and Vaccine Development, Ministry of Health and Social Welfare, Dar es Salaam, Tanzania; ^4^ RTI International, Washington, District of Columbia; ^5^ Preventive Services, Ministry of Health and Social Welfare, Dar es Salaam, Tanzania; ^6^ ENVISION, IMA WorldHealth, Dar es Salaam, Tanzania

## Abstract

Global health practitioners are increasingly advocating for the integration of community-based health-care platforms as a strategy for increasing the coverage of programs, encouraging program efficiency, and promoting universal health-care goals. To leverage the strengths of compatible programs and avoid geographic and temporal duplications in efforts, the Tanzanian Ministry of Health and Social Welfare coordinated immunization and neglected tropical disease programs for the first time in 2014. Specifically, a measles and rubella supplementary vaccine campaign, mass drug administration (MDA) of ivermectin and albendazole, and Vitamin A were provisionally integrated into a shared community-based delivery platform. Over 21 million people were targeted by the integrated campaign, with the immunization program and MDA program reaching 97% and 93% of targeted individuals, respectively. The purpose of this short report is to share the Tanzanian experience of launching and managing this integrated campaign with key stakeholders.

The neglected tropical diseases (NTDs) contribute to extensive disease and disability globally. The United Republic of Tanzania is endemic for all five NTDs for which preventive chemotherapy via mass drug administration (MDA) is standard of care. In 2013, over 40 million Tanzanians required MDA for onchocerciasis, lymphatic filariasis (LF), soil-transmitted helminths (STHs), schistosomiasis, or trachoma.^1^ The proportions of the population targeted and successfully reached by MDA campaigns in 2013 were 99%, 86%, 64%, 56%, and 82% for onchocerciasis, LF, STH, schistosomiasis, and trachoma, respectively (Ministry of Health [MOH], unpublished data). Integration of NTD programs may be an effective strategy for reaching global NTD control and elimination benchmarks, the given evidence suggesting that NTD integration is associated with increased coverage and reduced costs.^2^–^4^

Vaccination campaigns delivered according to the Expanded Program on Immunizations (EPI) schedule have consistently attained high coverage in Tanzania due to the availability of funding from the Gavi Vaccine Alliance and the launch of the Reaching Every Child approach. EPI services in Tanzania target children under 24 months of age with a nine-vaccine package, and in 2011, EPI programs integrated the distribution of mebendazole to children under 5 years of age for STH control. Some vaccine-preventable illnesses such as measles and rubella (MR) require supplementary campaigns every 3 years to ensure that epidemics do not occur in areas with low coverage or inadequate seroconversion rates.

Although experts suggest that NTD programs consider coordinating with other community-based public health programs to maximize coverage and efficiency, community-wide MDA and EPI campaigns typically operate independently.^5^,^6^ However, in 2014, temporal and geographic congruencies in Tanzania allowed the programs to provisionally coordinate the delivery of MDA and immunization supplementation campaigns for the first time. The purpose of this report is to share the experiences of the Tanzanian MOH in launching and managing this integrated campaign with relevant stakeholders.

The coordinated campaign was conceived during a weekly preventive services meeting in 2014, when NTD and immunization program managers were planning interventions with similar targeted age groups in many congruent geographies. The immunization program was scheduled to deliver a supplementary MR vaccination campaign to children 9 months to 15 years of age along with mebendazole for children under 5 years of age. The NTD MDA campaign was scheduled to distribute ivermectin and albendazole to individuals over 5 years of age for the treatment of onchocerciasis, LF, and STH. The MR campaign targeted 27 of the country's implementation units, with 16 of the areas also targeted by the NTD campaign. Thus, the hypothesis driving the integrated campaign was that coordinated delivery in cotargeted areas would efficiently use limited resources and, relative to vertical campaigns, increase coverage.

Activities conducted in coordination with one another included planning exercises, community sensitization and media campaigns, codistribution of drugs and vaccines, and monitoring and evaluation. The codistributed interventions targeted over 21 million people and are depicted in Figure 1

**Figure 1.**
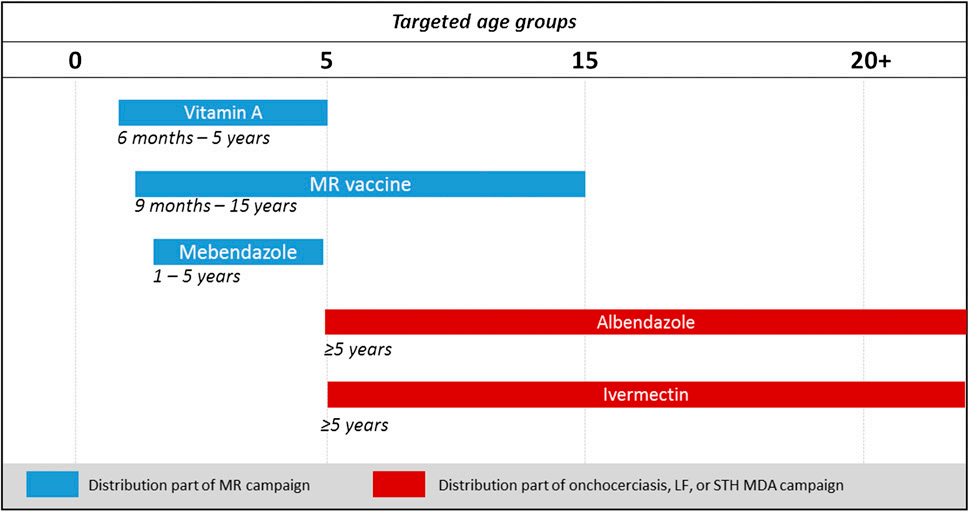
Interventions and targeted age groups of the Tanzanian coordinated measles and rubella (MR) and mass drug administration (MDA) campaign.

.

To facilitate delivery, 12,824 stationary and mobile vaccine posts were established. Each health post required seven people, including two health-care workers providing immunization services, two community drug distributors (CDDs) conducting MDA, two health workers (often school teachers) serving as data recorders, and one community leader mobilizing communities. A total of 1,886 council team supervisors were deployed to supervise the integrated teams.

The Tanzanian immunization program utilizes a “proxy system” whereby the population size and the number of people targeted are used to calculate daily coverage benchmarks. Daily benchmarks outline the number of days it will take to fully immunize the target population in each geography, and the number of people to reach per day. This system was adopted by the coordinated MR-MDA campaign, helping both programs to estimate the materials needed and the number of fixed or mobile teams required.

The coordinated campaign lasted for 7 days, followed by program-specific mop-up activities. In comparison, the 2013 MDA campaigns were conducted from August to December, typically spanning 7 days each in any given district. The 7 days were intensive, requiring daily travel and communication between health workers and supervisors. Coverage data from health posts were transferred to the national level daily. This was important in that remedial action was taken when daily coverage benchmarks were not reached. For example, if coverage was low due to a shortage of medications, it could be addressed within 24 hours, rather than at the end of the campaign as customary during routine MDA implementation.

Favorable changes in the primary outcome of interest, that is, program coverage, were observed. Routinely reported program coverage (i.e., the number of people reached divided by the number of people targeted) of the LF MDA program increased from 86% in 2013 to 93% in 2014 following additional mop-up activities. MR vaccination coverage remained high with 97% coverage in 2014 relative to the previous campaign in 2011 that achieved 96% coverage (Figure 2

**Figure 2.**
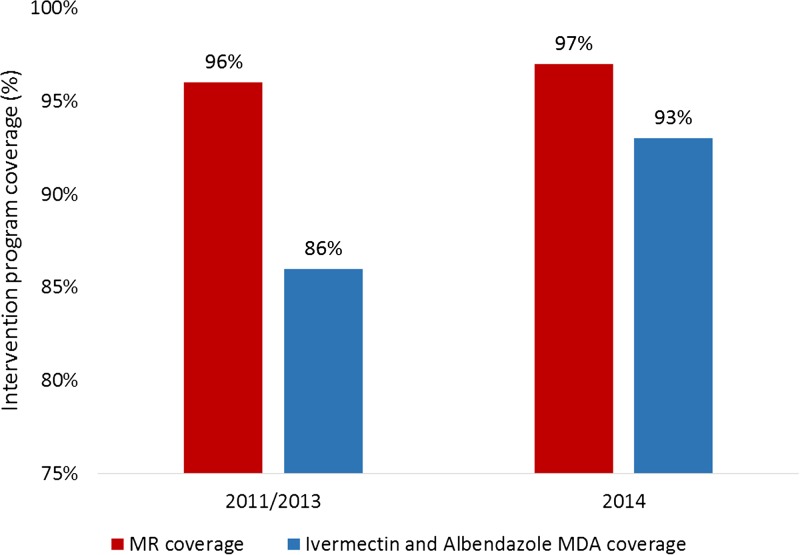
Measles and rubella (MR) and mass drug administration (MDA) program coverage in 2011/13 and 2014.

). An MR mop-up campaign was required in areas that were not NTD-endemic and were not included in the coordinated campaign.

It is likely that the joint campaign increased coverage relative to previous vertical campaigns by reducing the amount of time community members expended on community-based health-care activities and increasing the perceived incentives associated with a single health-care event. In addition, there is high demand for immunizations in the communities due to years of Gavi advocacy, and it benefited the MDA program to be linked to these popular services. However, MDA coverage estimates are influenced not only by the accuracy of demographic data (i.e., ratio denominators), but also by the accuracy of CDDs in reporting the number of people treated during a campaign. Because the health workers involved in the coordinated campaign had more extensive training than CDDs involved in vertical MDA campaigns, it is possible that the data collected during the coordinated campaign were more accurate than that reported during previous vertical MDA campaigns.

The secondary outcome of interest, that is, program costs, may have increased with the coordinated campaign. Macro costing budgets were prepared at the district level, identifying cost inputs such as personnel allowances, transportation fuel costs, supplies, etc. The total budgeted cost of implementing the last round of both programs separately (measles supplementary campaigns in 2011 and MDA in 2013) was US$6.04 million, whereas in 2014, the total joint cost was US$7.19 million. This observed increase in budgeted financial costs is probably due to a number of factors: rubella vaccines were not offered jointly with measles in 2011, a wider range of age groups were provided services in 2014 relative to earlier years, Vitamin A was not offered jointly with immunizations in 2011, and there are unique start-up costs associated with launching a newly integrated program. It was not possible to compare the disaggregated expenditures of the campaigns given that itemized activity and input costing exercises were not conducted.

There were a number of challenges to coordinated implementation, as identified during daily feedback meetings with districts mid-campaign and postcampaign debriefs. Because MDA was not conducted in all areas targeted by MR campaigns, the immunization program had to conduct separate immunization campaigns in 11 areas. In addition, per diems from NTD MDA campaigns are an income source for CDDs; however, few were involved in the coordinated MR-MDA campaign due to vaccine delivery training requirements. To counter any resulting loss to CDD retention, the MOH required different CDDs to be involved on different days to allow more volunteers to participate and benefit from daily per diems. In addition, many of the CDDs were engaged in community mobilization rather than intervention delivery. Accordingly a significant proportion of joint funds were proactively set aside for mobilization activities.

To our knowledge, this is the first national-level program to coordinate immunization activities with MDA for LF and STH, and there were a number of lessons learned relevant to other coendemic country governments and partners. First, Gavi activities are highly amenable to multisectorial coordination as integrated community-based activities are valued by the organization. Second, integration of child survival interventions minimized redundancies by removing repeat activities (e.g., health worker supervision) in the same target populations in the same time period. Third, leveraging the existing infrastructure by conducting activities in a mix of health-care posts including schools, health facilities, and community vaccination posts was critical in reaching all target populations. Fourth, reviewing data quality and completion at the end of each day was time consuming for supervisors but allowed for problems to be addressed immediately and rectified without delay. Most importantly, the programs found that coordinated coimplementation is a potential and often overlooked solution for increasing the coverage of community-based programs without compromising quality. This coordinated approach is also important for health systems strengthening, where cross-sector activities can be used to drive broader system improvements.

The primary limitation of this work is that routine outcomes and data collection measures were used to assess intervention efficiency. With the addition of a rigorous research protocol, the intervention could have been tested using a multistage rollout scheme and counterfactual. Future iterations of this intervention will use such methods, and it is our hope that other endemic countries and partners interested in coordinated community-based activities learn from these experiences.
